# Understanding Attrition in Text-Based Health Promotion for Fathers: Survival Analysis

**DOI:** 10.2196/44924

**Published:** 2023-08-18

**Authors:** Richard Fletcher, Casey Regan, Jason Dizon, Lucy Leigh

**Affiliations:** 1 School of Health Sciences College of Health, Medicine, and Wellbeing The University of Newcastle Callaghan Australia; 2 Hunter Medical Research Institute New Lambton Heights Australia

**Keywords:** attrition, dropout, text-based program, parenting, fathers

## Abstract

**Background:**

Web-based interventions targeting parents with health and parenting support frequently report high rates of attrition. The SMS4dads text messaging program, developed in Australia, has delivered texts to over 10,000 fathers. The brief text messages, which are sent 3 times per week from 16 weeks of gestation to 48 weeks after birth, include regular reminders that participants can leave the program by texting back “STOP” to any message. Although acceptance of the program is high, almost 1 in 5 ask it to be removed. Analyzing the factors influencing attrition from digital parenting programs such as SMS4dads may assist in developing more effective interventions.

**Objective:**

This study aimed to examine factors associated with attrition in a text-based intervention targeting fathers.

**Methods:**

Demographic characteristics, requests to complete a psychological scale, individual message content, participant feedback, and automatically collected data registering clicks on links embedded in the texts were examined to identify attrition factors among 3261 participants enrolled in SMS4dads from 4 local health districts in New South Wales, Australia, between September 2020 and December 2021.

**Results:**

Participants who were smokers, recorded risky alcohol consumption, had a lower education level, or signed up prenatally had 30% to 47% higher hazard of dropout from the program, whereas participant age, Aboriginal or Torres Strait Islander status, rurality, and psychological distress score (as Kessler Psychological Distress Scale [K10] category) were not associated with dropout. Primary reasons for dropping out reported by 202 of 605 respondents included “other reasons” (83/202, 41.1%), followed by “not helpful” (47/202, 23.3%) and “too busy” (44/202, 21.8%). Program features such as repeated requests to complete a psychological scale (K10) and the content of individual messages were not linked to increased dropout rates. Analysis of a sample (216/2612) of inactive participants who had not engaged (clicked on any embedded links) for at least 10 weeks but who had not opted out identified a further 1.5% of participants who would opt to leave the program if asked.

**Conclusions:**

Identifying which features of the participant population and of the program are linked to dropout rates can provide guidance for improving program adherence. However, with limited information from feedback surveys of those exiting early, knowing which features to target does not, by itself, suggest ways to increase engagement. Planning ahead to include robust measures of attrition, including more detailed feedback from participants, could provide more effective guidance. A novel element in this study was seeking feedback from inactive participants to estimate dropout from this group and thereby provide an overall dropout rate of 20%. The retention rate of 80%, relatively high compared with other web-based parenting programs for fathers, suggests that tailoring the content to specifically address fathers’ role may be an important consideration in reducing fathers’ disengagement.

## Introduction

Over recent years, parenting programs’ move to text-based or web-based digital versions of what previously were face-to-face group programs has accelerated [1]. Digital programs, which obviate the need for and expense of travel and fixed attendance times, have the potential to reach parents who are widely dispersed and who have multiple commitments in their daily lives.

However, although the acceptability of digital educational health- and parenting-related resources appears to be high, retaining participants once enrolled has been a challenge [1,2]. Online parenting programs are easily accessed but participants can also easily withdraw [3], and dropout rates of over 90% have been reported [4]. High rates of attrition are a concern for program validity [5] but are often underreported in published studies of online health-related interventions. Calls have been made for a “science of attrition,” accepting that interventions will not be universally appropriate and reporting on the multiple factors that can be linked to discontinuation [6-8]. The assessment of attrition in the mental health and health promotion fields has moved beyond measuring the number of online modules completed or the number of clicks on a website [9,10]. The attrition concept has been linked to engagement, which may involve emotional, cognitive, and behavioral measures and the notion that more engagement does not necessarily equate to better results, suggesting that the levels of optimal engagement or dosages are important to measure [11-13].

Specifying the optimal level of engagement requires the ability to identify those who are “nonadherers,” participants who decide not to follow the recommended level of participation for their own reasons, or “nonusers,” who disengage from the material even while they continue to be enrolled [6,11]. Nonuse may constitute a proxy for attrition due to both reflecting participants’ “losing interest” in the program; however, it may be also a lapse in interest if, in programs extending for months, participants may return to engage after a period of absence [12,13]. For text-based parenting programs, where messages are sent as SMS text messaging notifications, whether recipients open or read the message is not easily established. Documenting participants’ actions when they access supplementary material via website addresses embedded in the texts can provide a measure of not only continued enrollment but also engagement with the web-based program [14].

The reasons for participants withdrawing from health promotion and mental health digital interventions, ranging from demographic variables (age and education level) and program features (relevance and participant burden) to contextual factors (changing circumstances and internet connection), are rarely fully explored [8,15]. Analysis of attrition in fathers is particularly relevant as they are regarded as both “hard to reach” [16] and are more likely than mothers to leave parenting and eHealth programs before completion [17]. In this paper, the retention of participants enrolled in the SMS4dads program [18] for men transitioning to fatherhood is examined to describe measures of attrition that may be suitable for text-based parenting and mental health support programs.

## Methods

### Setting

The Focus on New Fathers pilot [19] delivered brief plain language texts to soon-to-be and new fathers that contain tips, prompts, information, and links to web support—SMS4dads. NSW Health promoted the availability of SMS4dads via social and paid media. Participants (fathers) from 4 local health districts in the state of New South Wales, Australia, were able to enroll in SMS4dads at any time from 16 weeks of gestation until 24 weeks after birth and receive messages (approximately 3 per week) until 48 weeks after birth. The messages, which were limited to 160 characters and a reading age of 7 years (Flesch-Kincaid level <9), provided tips that were timed to match the development of the fetus or baby and were designed to encourage father-infant engagement, father-partner support, and self-care. Approximately 30% of texts included a hyperlink to not-for-profit mental health or parenting websites with further information. At enrollment and at 3 subsequent points in the schedule of messages, participants were invited to complete the Kessler Psychological Distress Scale (K10) [20]. A purpose-designed Mood Checker interactive text was sent at 3 weekly intervals. Mood Checker messages were keyed to common challenges faced by parents during pregnancy and after birth, such as infant crying or reestablishing intimacy. Participants who indicated that they were distressed (high score on the K10 or response to interactive Mood Checker text that they were distressed and “wanted help now”) were linked to online professional mental health support. Although the program resulted in over 3000 fathers enrolling, an important measure of the success of the program was the ability of SMS4dads to retain participants in the service to keep receiving the texts.

Participants could leave the program at any point by texting back “STOP” or similar words to any message. When such a reply was received, SMS4dads staff manually activated the request using a password-protected webpage to remove the mobile number from all SMS4dads active elements. An automated message was then sent to the participants advising them that messages had stopped and asking them to provide a reason for their dropout request.

### Data Collection for Attrition Analysis

Data collected for the attrition analysis included demographics, exit survey data, data related to 2 program features, and data on inactive participants.

#### Demographic Data

At enrollment, through the SMS4dads website, participants entered their age, baby’s date of birth (or expected date of birth), Aboriginal or Torres Strait Islander status, residential address, educational level, smoking status, alcohol consumption (based on the Alcohol Use Disorders Identification Test-Concise; AUDIT-C) [21], and whether their enrollment was for their first child; they also completed the K10 questionnaire.

#### Exit Survey

When participants texted “STOP” to SMS4dads, an automated message was sent asking them to provide a reason for their dropout request (1=“not helpful,” 2=“I did not sign up for this,” 3=“situation has changed,” 4=“too busy,” and 5=“other reasons”).

#### Program Feature Identification

Two features of the SMS4dads program, message content and K10 questionnaire invitations, were identified as possible triggers of dropout.

##### Message Content

Program messages (160 characters maximum) were designed to speak frankly to soon-to-be and new fathers offering tips and information requiring moderate (Flesch-Kincaid level <9 [22]) literacy level. It is possible that some messages suggesting paying attention to the infant or thinking about the needs of the mother would be off-putting or irritating as they may seem to criticize the father as inattentive or uncaring. To identify possible content triggers for dropping out, the unique message delivered immediately before a dropout request was recorded.

##### K10 Questionnaire Invitation

It is also possible that asking participants to complete a psychologically oriented questionnaire (K10) may have seemed intrusive or too demanding and led to participants withdrawing from the program [8]. Completion of the K10 at enrollment was mandatory, and once messaging began, participants were invited again to complete the scale once prenatally (conditional on receiving at least 4 weeks of messages) and 3 times after birth. To investigate whether K10 questionnaire invitations possibly increase the likelihood of dropout, the number of days between the most recent K10 invitation and a “STOP” request from the participant was recorded.

#### Inactive Participants’ Identification

Inactive participants are those who remain enrolled in the SMS4dads program and continue to receive text messages but do not engage. Because the text messages sent to participants’ mobile phones did not require a login or response, participants were considered to be engaged when they clicked on the embedded website links within the messages or in the interactive Mood Checker texts. Clicks were automatically logged by the server software on the website.

From interview responses reported in an earlier pilot study of SMS4dads [23], it was apparent that new fathers experienced periods of competing demands across family, work, and relationships when they may be unable to read or respond to messages. Also, approximately 22% of those enrolled scored above the threshold for symptomatic depression on the K10, which included the question “During the last 30 days, about how often did you feel that everything was an effort?” It was assumed that a short period of nonresponse to text messages might indicate a temporary lack of engagement rather than signaling that the participant had ceased viewing the texts.

**Figure 1:**
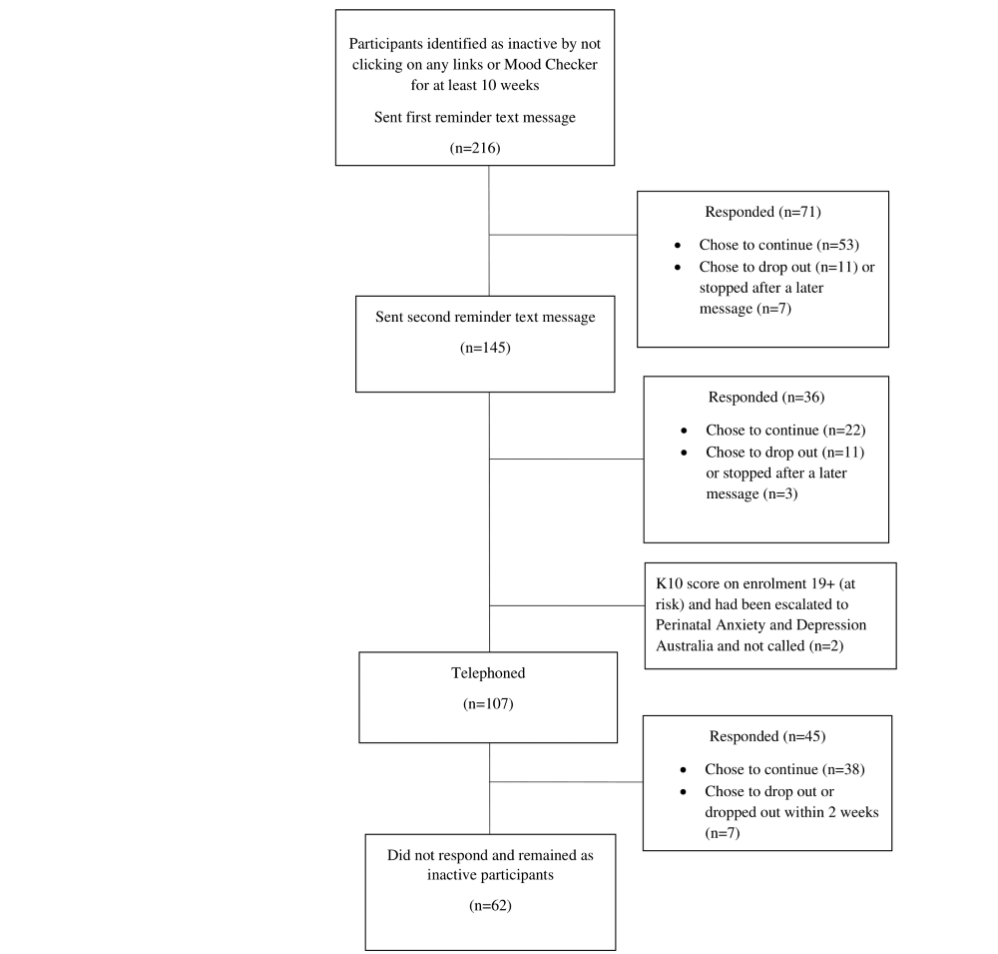
Flow diagram of inactive participant identification.

To distinguish transient disengagement from inactivity, participants who had not dropped out but who had also not clicked on any links or the Mood Checker link for at least 10 weeks in May 2021 were identified. These participants were sent a courtesy text advising them that links had not been clicked recently and asking them to confirm whether they wanted to continue participation by texting back a response. Those failing to reply were sent a second reminder text message. Those not replying to the second reminder were telephoned to ask if they still wanted to receive the messages. Calls were conducted during business hours, and when the call was directed to an answering service, a message was left reminding participants that they could text back “STOP” to any message if they wished to drop out. The inactive participant identification process is described in Figure 1.

### Ethics Approval

The study received approval from the Human Research Ethics Committee of the University of Newcastle, New South Wales (approval number H-2016-0055). Participants provided informed consent during registration to the program and received an information statement explaining the potential use of their data for research activities, allowing secondary analysis without additional consent. To protect the privacy of participants, some data were deleted during extraction, such as the participants’ name, address, and contact information. To the researchers, the participants were anonymous or unidentified. No compensation was received by participants.

### Statistical Approach

#### Characteristics of Participants

The association between each baseline characteristic (rurality, Aboriginal or Torres Strait Islander status, education level, perinatal stage at sign-up, smoking status, alcohol risk using AUDIT-C, K10 distress score level, whether it was their first child, and participant age) and dropping out from the program was examined for all participants using Cox regression. Estimates from the survival analysis are presented as hazard ratios (HRs) with 95% CIs.

#### Program Features

##### Message Content

Frequencies and percentages are reported for the messages most frequently preceding a dropout request. As messages directly aligned with the baby’s date of birth (or expected date of birth), survival probability was examined using a survival curve with gestational age as the unit of time. This curve was created from a Cox model using a counting process style of input to account for differing gestational ages upon entry to the study.

##### K10 Questionnaire Invitation

A comparison between dropout within 1 to 2 weeks after a K10 questionnaire invitation and all other weeks was made using a pooled logistic regression. The models included a variable for a week in the study and variables indicating whether this week was within 1 week of K10 invitation or within 2 weeks of K10 invitation, modeling the probability that the participant dropped out during a given week. Model estimates are presented as odds ratios (ORs) with 95% CIs and *P* values.

### Exit Survey

Frequencies for all reasons for dropping out are presented. Chi-square tests compared differences among the following demographic variables between participants who completed the exit survey (ie, provided a reason for dropping out) and those who did not complete the exit survey: rurality (rural or urban), Aboriginal or Torres Strait Islander status, education level (bachelor degree or above, advanced diploma or diploma, certificate III/IV, year 12, and year 11 or below), perinatal stage at sign-up (prenatal or postnatal), smoking status (smoker or nonsmoker), AUDIT-C (not at risk or at risk), and K10 distress score (low, 10-15; moderate, 16-21; high, 22-29; and very high, 30-50).

## Results

Between September 2020 and December 2021, of the 3261 participants who enrolled, 605 (18.6%) requested to exit the program before reaching the final text sent at 48 weeks after birth.

### Demographic Associations With Dropout

Table 1 shows descriptive statistics of those who exited before completion. Participants who dropped out were enrolled for an average of 17.4 (SD 17.1) weeks and received an average of 66 (SD 64) texts. In terms of baby age, participants sent dropout requests from 25 weeks before the expected due date through to 53 weeks after birth (mean 9; SD 14 weeks).

Table 2 shows the crude and adjusted HRs for each baseline characteristic of interest. The sample considered by the models included all participants. The adjusted model used 3229 observations. Compared with their counterparts, participants who were smokers, recorded risky alcohol consumption, had a lower education level, or signed up prenatally had 30% to 47% higher hazard of dropout from the program. Additionally, compared with participants expecting their first child, participants who were expecting another child had 82% higher hazard of dropout from the program (HR 1.82, 95% CI 1.48-2.23; *P≤*.001). Participant age, Aboriginal or Torres Strait Islander status, rurality, and psychological distress score (K10 category) were not associated with dropout.

**Table 1:** Characteristics of dropout requests (n=605).

Continuous variables		Mean (SD)	Median	Minimum	Maximum
**Baby characteristics**					
	Baby’s age when dropout requested (weeks)	9 (14)	7	−25	53
**Program characteristics**					
	Messages sent before dropout request	66 (41)	64	1	232
	Time enrolled before dropout request (weeks)	17.4 (10.9)	17.1	0	45
**K10^a^ invitation or reminder characteristics (n=322)**					
	Time from K10 invitation before dropout request until dropout request (days)	56 (37)	57	0	125

^a^K10: Kessler Psychological Distress Scale.

**Table 2:** Association between dropout and baseline characteristics.

Characteristic/response			Crude				Adjusted			
			HR^a^ (95% CI)	*P* value		HR (95% CI)		*P* value		
**Rurality (n=** **3242** **)**										
	Urban (n=493)	Reference		—^b^	Reference				—	
	Rural (n=2749)	1.32 (1.08-1.62)		.008	1.10 (0.88-1.38)				.40	
**Aboriginal or Torres Strait Islander (n=** **3246** **)**										
	No (n=3189)	Reference		—	Reference				—	
	Yes (n=57)	1.52 (0.91-2.54)		.11	1.17 (0.68-2.01)				.57	
**First child (n=** **3259** **)**										
	Yes (n=2763)	Reference		—	Reference				—	
	No (n=496)	1.60 (1.32-1.95)		<.001	1.82 (1.48-2.23)				<.001	
**Smoke (cigarettes or tobacco; n=** **3259** **)**										
	No (n=3051)	Reference		—	Reference				—	
	Yes (n=208)	1.51 (1.14-1.99)		.004	1.45 (1.06-1.99)				.02	
**Education (n=** **3259** **)**										
	Bachelor’s degree or above (n=2277)	Reference		—	Reference				—	
	Advanced diploma or diploma (n=253)	1.52 (1.16-1.99)		.002	1.44 (1.10-1.90)				.009	
	Certification III/IV (n=430)	1.38 (1.11-1.73)		.004	1.24 (0.98-1.57)				.07	
	Year 12 (n=191)	1.51 (1.11-2.05)		.009	1.41 (1.03-1.94)				.03	
	Year 11 or below (n=108)	1.58 (1.07-2.33)		.02	1.31 (0.85-2.02)				.22	
**Perinatal stage at sign-up (n=** **3261** **)**										
	Prenatal (n=1973)	Reference		—	Reference				—	
	Postnatal (n=1288)	0.74 (0.62-0.87)		<.001	0.70 (0.59-0.83)				<.001	
Father’s age (n=3261)			0.99 (0.98-1.01)	.37		0.99 (0.98-1.01)				.40
**AUDIT-C^c^ category (n=3259)**										
	Score <4 (n=2305)	Reference		—	Reference				—	
	Score ≥4 (n=954)	1.40 (1.19-1.65)		<.001	1.47 (1.23-1.75)				<.001	
**K10^d^ category (n=3261)**										
	Low (n=1778)	Reference		—	Reference				—	
	Moderate (n=953)	0.91 (0.76-1.09)		.31	0.89 (0.74-1.07)				.23	
	High (n=392)	1.00 (0.78-1.29)		.99	0.92 (0.71-1.18)				.51	
	Very high (n=138)	1.02 (0.68-1.52)		.93	0.87 (0.57-1.31)				.49	

^a^HR: hazard ratio.

^b^Not available.

^c^AUDIT-C: Alcohol Use Disorders Identification Test-Concise.

^d^K10: Kessler Psychological Distress Scale.

### Program Features Associated With Dropout

#### Message Content

Identifying the message received by participants immediately before sending a “STOP” request does not indicate that specific messages are particularly involved in participants’ decisions to leave the program. Most of the messages (176/240) were followed by at least 1 participant requesting to exit. Those messages with higher frequencies (≥10) are included in Table 3 according to domain. As can be seen, 8 of those 9 messages include the prompt “[Txt STOP to OptOut],” which is added to approximately every 10th message reminding participants that they can leave the program simply by texting “STOP” in response to a message. The frequency of instructions on how to leave a text-based program targeting mothers has been linked to rates of attrition [24]. As can be seen from Table 3, the most prevalent message before which participants requested to drop out was the text “4dad: It can be tough to leave your partner and baby to go to work...,” which was sent approximately 9 weeks after birth.

**Figure 2:**
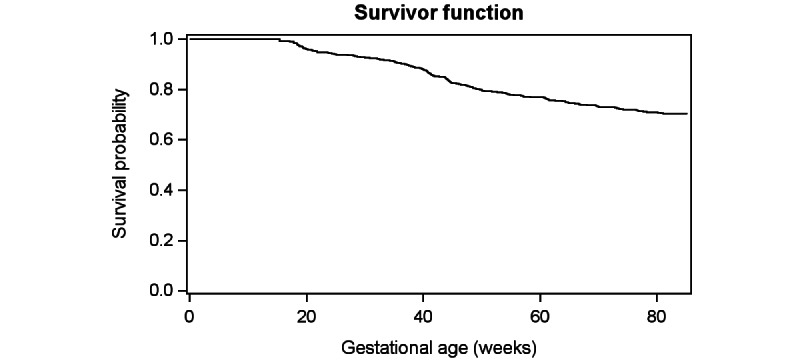
Survival by gestational age.

Another possible reason for the higher than average number of stop requests, besides the message content, may also be the gestational age with which the message aligns. Figure 2 shows the survival curve with respect to gestational age. The curve shows the probability of a participant staying in the study (surviving) as the gestational age of their child increases. There did not appear to be any gestational age where the trajectory of the survival probability exhibited a rapid change (ie, the rate of dropout seemed to be constant for gestational age).

**Table 3:** Most common last received messages before the dropout request (n=605).

Domain and text message		Texts, n (%)
**Father-partner support**		
	4dad: It can be tough to leave your partner and baby to go to work. Maybe texting can keep you in touch. [Txt STOP to OptOut]	50 (8.3)
	4dad: This is a time when many parents worry about a lot of things. Talking about your worries with your partner can really help you both. [Txt STOP to OptOut]	17 (2.8)
	4dad: Find ways to tell your partner she is doing an amazing job. This could be really important to her. [Txt STOP to OptOut]	14 (2.3)
	4dad: Breastfeeding is natural but doesn’t come naturally to many mums. Your support will be important for her no matter how it works out. Dads guide to BF here <<link removed>>	10 (1.7)
**Father-infant attachment**		
	4dad: Hi dad. If I am unsettled then doing something active inside or out can be good for both of us. [Txt STOP to OptOut]	13 (2.1)
**Self-care**		
	4dad: I am going to triple my weight in the first year of life. Don’t let this happen to you too dad. [Txt STOP to OptOut]	22 (3.6)
	4dad: Catching up with family and friends is good for baby and good for parents. [Txt STOP to OptOut]	18 (3)
	4dad: It could be time to think about connecting with some old friends. Have things settled down enough for that yet? [Txt STOP to OptOut]	15 (2.5)
	4dad: Are you thinking about the kind of dad you want to be? Focus on all the positive things you have done and can do in the future. [Txt STOP to OptOut]	13 (2.1)

#### K10 Questionnaire Invitation

Participants requested to drop out any time from zero to 125 days after receiving a K10 questionnaire invitation (mean 56, SD 37; Table 1). Table 4 shows the association between dropout and being sent an invitation to complete a K10 questionnaire. Although the odds of dropping out within 1 week after a K10 invitation were 15% higher (OR 1.15, 95% CI 0.79-1.68; *P*=.47) compared with all other weeks, and the odds of dropping out within 2 weeks after a K10 invitation were 7% higher (OR 1.07, 95% CI 0.81-1.43; *P*=.63) compared with all other weeks, neither result was statistically significant.

**Table 4:** Odds of dropping out 1 and 2 weeks after receiving an invitation to complete a K10^a^ questionnaire.

Variable	OR^b^ (95% CI)	*P* value
1 week after K10 invitation	1.15 (0.79-1.68)	.47
2 or more weeks after K10 invitation	1.07 (0.81-1.43)	.63

^a^K10: Kessler Psychological Distress Scale.

^b^OR: odds ratio.

### Reasons for Dropping Out

Of all participants who were sent an exit survey, approximately one-third replied (202/605, 33.4%) with a reason for dropping out. The reasons for the dropout request are given in Table 5.

Chi-square tests indicated that there were no significant differences in baseline demographic characteristics between participants who responded to the exit survey and those who did not, including rurality, Aboriginal or Torres Strait Islander status, education level, perinatal stage at sign-up, smoking status, AUDIT-C risk, and K10 distress score.

**Table 5:** Reasons for the dropout request (n=202).

Reason reported	Reason, n (%)
Did not sign up for this	5 (2.5)
Situation changed	23 (11.4)
Too busy	44 (21.8)
Not helpful	47 (23.3)
Other reasons	83 (41.1)

### Inactive Participants

Of the 2612 participants currently enrolled who had not opted out, 216 had not clicked on any links or the Mood Checker link for at least 10 weeks in May 2021 and were sent a reminder text message (Figure 1). One-third of participants (71/216, 32.9%) provided a response indicating that they wanted either to continue in the program (53/71, 74.6%) or wanted to drop out (18/71, 25.4%). The remaining participants were sent a second reminder message, to which one-quarter (36/145, 24.8%) responded (22/36, 61.1% opted to continue and 14/36, 38.9% opted to drop out), leaving 109 participants to be telephoned. A total of 45 participants who were called answered or responded to the voicemail message (“checking if you want to continue”), with most choosing to continue in the program (38/45, 84.4%) and others opting to drop out (7/45, 15.6%), leaving 64 participants who made no response during this process and did not take action to drop out in response to the phone message reminder. Among those who responded, some participants offered explanations for inactivity, such as “a busy period at work” or “I never click on links because of scams.” Several stressed that they valued the messages even though they did not click links.

In sum, of the participants who had not clicked on any links or Mood Checker for at least 10 weeks, 52.3% (113/216) confirmed that they wanted to remain in the program, 28.7% (62/216) remained in the program but did not indicate their intention, and 18% (39/216) chose to drop out. Applying this ratio of inactive users (216/2612) to the total participants (n=3261) suggests that 270 participants would be inactive users. Of these, 49 (18%) could be expected to drop out, indicating a total estimated dropout rate of 20.1%.

## Discussion

### Principal Findings

Assessing the attrition of participants is an important feature of program evaluation and improvement. In this analysis, 4 approaches were adopted to identify possible key features of attrition, which may, in turn, provide guidance for participant retention in text-based parenting or mental health interventions. When demographic factors were analyzed, several factors were identified that do not appear to influence retention: age, living in a rural area, being Aboriginal or a Torres Strait Islander, or experiencing high levels of psychological distress. However, men enrolling postnatally, men with previous children, smokers, men without tertiary education, and men consuming alcohol at risky levels were found to be more likely to exit before completion.

The increased attrition for experienced fathers is understandable, as they may have previously encountered the information on infant development that is provided in SMS4dads. However, for those enrolling before the birth, the higher numbers exiting before the final texts at 48 weeks may reflect participant fatigue; possibly, the participants did not find the messages stimulating after many months. Because smoking and alcohol behaviors are not mentioned in the text messages, the increased attrition is unlikely to be linked to specific content of the program but may reflect characteristics of substance users such as impulsivity [25] or be linked to lifestyle factors [26]. Those with lower educational attainment, who are also more likely to smoke and be at risk in their alcohol consumption, may also be negatively influenced by lifestyle factors. A digital intervention targeting men from low-income areas found that the rate of attrition was higher in men from “high-risk” neighborhoods than from “resilient” neighborhoods [27].

Both smoking and alcohol consumption could be the subject of texts for fathers, as these behaviors are recognized as potential risks to the health and well-being of pregnant women and young infants [28,29]. However, even though programs specifically targeting fathers’ smoking and alcohol use have been reported [30,31], the branding of SMS4dads and message content is designed as “tips and information” for new fathers, not as a resource for “fathers who have a problem,” and so any additional messages for fathers on these topics may require an “opt in” process. The education level of participants may be better accommodated by reducing the literacy demands of the texts, and the postnatal texts may be reviewed to maintain interest among those participants who enrolled prenatally.

Two aspects of the SMS4dads program were investigated as possible triggers for participants to leave: being asked to complete the K10 scale and receiving particular messages. The analysis found that there was no significant increase in participants’ likelihood of exiting within 1 or 2 weeks of receiving the invitation to complete the K10. A frequency count of messages that occurred immediately before participants requested “STOP” revealed that some messages had higher rates of subsequent dropout, but no discernible pattern was evident apart from the common feature of a reminder to “opt out.” The message with the highest linked dropout number occurred at 9 weeks after birth. As discussed by Saleem et al [12], an approach to engagement that aims for “effective engagement” may be more appropriate to digital interventions than simply aiming to maximize engagement. The period after the birth, when particularly stressful challenges such as infant crying have peaked (after 6 weeks [32]), may be a period when fathers feel as if they are managing their family situation well enough that they do not require the text messages. In this case, an analysis of dropouts by baby age revealed no significant points of increased risk of dropout. However, a prerequisite to defining an optimum number of weeks for fathers to continue enrollment may be a clearer understanding of the needs of various subgroups of fathers in Australia.

Of those giving reasons for asking to be removed from the SMS4dads program, the low number of “not helpful” responses may indicate general agreement with the program content and delivery. However, for improving retention, having more detailed reasons for dissatisfaction would provide more guidance. Similarly, with only one-third of those exiting early providing any feedback response, and the most frequent reason provided being “other reasons,” the brief survey used in this study has not provided guidance for increasing retention or improving the content of the program. A more detailed exit survey, however, may not provide a useful measure of fathers’ engagement. Fathers and mothers expecting their first baby were recruited to the Baby Steps Wellbeing program, which consists of 9 web-based modules involving setting goals, solving problems, developing a plan, and taking action. Although only 21% of fathers used more than 1 module, compared with 54% of mothers, and only 13% of fathers set a goal, compared with 44% of mothers, scores for overall satisfaction, relevance, usefulness, and ease of finding what they wanted were not significantly different for mother and father participants [33]. Interviews have also been widely used to gain an understanding of disengagement [9]. However, interpreting the data obtained in the absence of guidelines or recognized standards in definitions of engagement has become difficult [9].

To investigate “inactive” participants, an arbitrary parameter of 10 weeks without clicking was chosen. Although other studies have used 30 days or 14 days without accessing the program to define nonuse, the extensive time frame for participation in SMS4dads, potentially 18 months if enrolled at 16 weeks, suggested a longer time measure. The process undertaken found that a subgroup of fathers, when contacted, asserted that they enjoyed the texts but had no interest in viewing the linked websites and resources. With this population in mind, a text-only version might be developed that would obviate the need for internet connection and use but that would require a different method of assessing active engagement in the program.

Overall, although demographic factors linked to exiting early were found and dropouts could be identified among inactive participants, the analysis of program features provided limited guidance for improving retention. Those designing “light touch” text-based health and parenting interventions may do well to plan their attrition analysis and include features that can give useful direction to improve retention.

The high level of participant retention in SMS4dads (80%) is in contrast to reported attrition rates in mental health and health promotion digital programs in general [2,34]. It is also markedly higher than programs promoted for parents that hoped to recruit and retain fathers in the intervention. An analysis of 28 Triple P research studies found that, in some studies, 100% of fathers dropped out [17]. The Baby Wellbeing program, which restricted recruitment to cohabiting couples, also failed to engage fathers and found only mothers benefited [33]. The ParentWorks program was heavily promoted to fathers with father-specific advertising but was offered as a generic parenting program to mothers and fathers. A total of 90% of enrolled fathers quit the program before completing the final module [4]. A feature of these programs is that, like almost all therapeutic and health promotion programs for parents, the content was trialed on mothers, and fathers were added as a target group only after efficacy was established with mothers [35]. By contrast, the content and language in the SMS4dads program is specifically tailored to fathers’ role during the perinatal period.

### Limitations

The SMS4dads program was designed as “light touch” so as to maximize program attractiveness to fathers. The study design therefore sought the minimum demographic data in the registration process and avoided the collection of data on the fathers’ experiences during the pregnancy, birth, and postnatal period. Characteristics of new fathers, such as unplanned pregnancy [36], experiencing a traumatic birth [37], or having a partner with mental distress [38], may have affected their commitment to their new role and, by extension, to the SMS4dads program. These factors were not assessed. The exit survey multiple-choice question on the “reason” for opting out, also designed with participant burden in mind, provided minimal guidance for improving retention.

### Conclusions

Analyzing program features can provide guidance for improving program adherence by identifying which features of the participant population and of the program are linked to dropout rates. Approaches to improving measurement of attrition have included scales assessing engagement [11,39] and acceptability [40], and several systematic reviews have provided overviews [1,5,34]. However, the variety of components included in defining and describing attrition have continued to expand, and the difficulty in comparing studies that assess differing parameters continues. In our analysis, demographic features that may be expected to increase attrition, such as rurality, elevated levels of distress, or age, were not related to dropout, nor were the 2 program features of psychological test requests and message content. Smoking and alcohol use were linked to dropout, and provision of texts targeting these groups may reduce disengagement. Also, reducing literacy demand and increasing stimulation level of postnatal texts may reduce dropout among low-education-level participants or those who enrolled prenatally. Seeking feedback from inactive participants allowed an estimate of dropouts among this group and thereby an overall retention rate of 80%. The SMS4dads program may provide guidance for other interventions seeking to engage fathers. Compared with programs offering self-paced online support for fathers using content designed to be equally appealing to fathers and mothers, the overall retention level of 80% is high. Future studies may usefully investigate if tailoring the content of “parenting” interventions to specifically address fathers’ role may reduce disengagement and attrition among male parents.

## Abbreviations

AUDIT-C: Alcohol Use Disorders Identification Test-Concise
HR: hazard ratio
K10: Kessler Psychological Distress Scale
OR: odds ratio

## Data Availability

The data sets generated during and/or analyzed during this study are available from the author on reasonable request.
